# Early rectal cancer: The diagnostic performance of MRI supplemented with a rectal micro-enema and a modified staging system to identify tumors eligible for local excision

**DOI:** 10.1177/20584601241241523

**Published:** 2024-04-18

**Authors:** Ellen Viktil, Bettina Andrea Hanekamp, Arild Nesbakken, Else Marit Løberg, Ole Helmer Sjo, Anne Negård, Johann Baptist Dormagen, Anselm Schulz

**Affiliations:** 1 Department of Radiology and Nuclear Medicine, Oslo University Hospital – Ullevål Hospital, Oslo, Norway; 2 Institution of Clinical Medicine, University of Oslo, Oslo, Norway; 3 Department of Gastrointestinal Surgery, 155272 Oslo University Hospital – Ullevål Hospital, Oslo, Norway; 4 Department of Pathology, Oslo University Hospital – Ullevål Hospital, Oslo, Norway; 5 Department of Radiology, Akershus University Hospital, Lørenskog, Norway

**Keywords:** rectal neoplasms, early diagnosis, magnetic resonance imaging, neoplasm staging, enema, lymphatic metastasis

## Abstract

**Background:**

In staging early rectal cancers (ERC), submucosal tumor depth is one of the most important features determining the possibility of local excision (LE). The micro-enema (Bisacodyl) induces submucosal edema and may hypothetically improve the visualization of tumor depth.

**Purpose:**

To test the diagnostic performance of MRI to identify ERC suitable for LE when adding a pre-procedural micro-enema and concurrent use of a modified classification system.

**Material and Methods:**

In this prospective study, we consecutively included 73 patients with newly diagnosed rectal tumors. Two experienced radiologists independently interpreted the MRI examinations, and diagnostic performance was calculated for local tumors eligible for LE (Tis-T1sm2, *n* = 43) and non-local tumors too advanced for LE (T1sm3-T3b, *n* = 30). Sensitivity, specificity, positive predictive value (PPV), and negative predictive value (NPV) were registered for each reader. Inter- and intra-reader agreements were assessed by kappa statistics. Lymph node status was derived from the clinical MRI reports.

**Results:**

Reader1/reader2 achieved sensitivities of 93%/86%, specificities of 90%/83%, PPV of 93%/88%, and NPV of 90%/81%, respectively, for identifying tumors eligible for LE. Rates of overstaging of local tumors were 7% and 14% for the two readers, and kappa values for the inter- and intra-reader agreement were 0.69 and 0.80, respectively. For tumors ≤T2, all metastatic lymph nodes were smaller than 3 mm on histopathology.

**Conclusion:**

MRI after a rectal micro-enema and concurrent use of a modified staging system achieved good diagnostic performance to identify tumors suitable for LE. The rate of overstaging of local tumors was comparable to results reported in previous endorectal ultrasound (ERUS) studies.

## Introduction

The diagnostic workup of early rectal cancer (ERC) relies on findings at endoscopy, digital rectal examination, biopsy, and imaging, with the depth of submucosal invasion as one of the most critical factors determining the possibility for local excision (LE) with curative intent. Currently, endorectal ultrasound (ERUS) is the imaging method of choice recommended by several guidelines.^1,2^ Meta-analysis investigating ERUS report on sensitivities for T1 tumors in the range of 50%–88% and specificities of 89%–98%.^3,4^ However, ERUS is a demanding examination with significant intra-observer variations, may be painful for the patient, not feasible in all patients, and not available in all hospitals due to lack of competent investigators or equipment.

Historically, MRI has focused on locally advanced tumors to define the need for neoadjuvant chemoradiotherapy, and MRI reports do often not provide sufficient information needed for treatment decisions for ERC.^4^

If preoperative investigations indicate a benign lesion or cancer with infiltration not exceeding the middle third of submucosa (Kikuchi T1sm1-sm2), patients in our hospital are treated with LE as an excisional biopsy.^5^ If the resection specimen shows no high-risk features for nodal disease as low differentiation or lymphovascular invasion at histopathology, LE is accepted as definitive treatment. We wanted to test a staging system adapted to these considerations with T1sm2 as the cutoff stage for accepting LE.

We routinely apply a micro-enema prior to rectal MRI examinations and have observed that the enema induces a widening of submucosa due to submucosal edema. Hypothetically, this could improve the visualization of tumor infiltration in submucosa and the staging of ERC.

The aim of this study was to test the diagnostic performance of MRI supplemented with a pre-procedural rectal micro-enema and concomitant use of a modified T-stage classification system to identify rectal tumors eligible for LE.

We also evaluated nodal disease since LE does not remove the nodes and positive nodal disease represents a critical restricting factor in the handling of ERC.

## Material and methods

### Patients

The study complies with the Declaration of Helsinki and was approved by the local Data Protection Official PVO and by the Regional Committees for Medical and Health Research Ethics (REC) in November 2015. Written informed consent was obtained from all patients.

**Figure 1.**
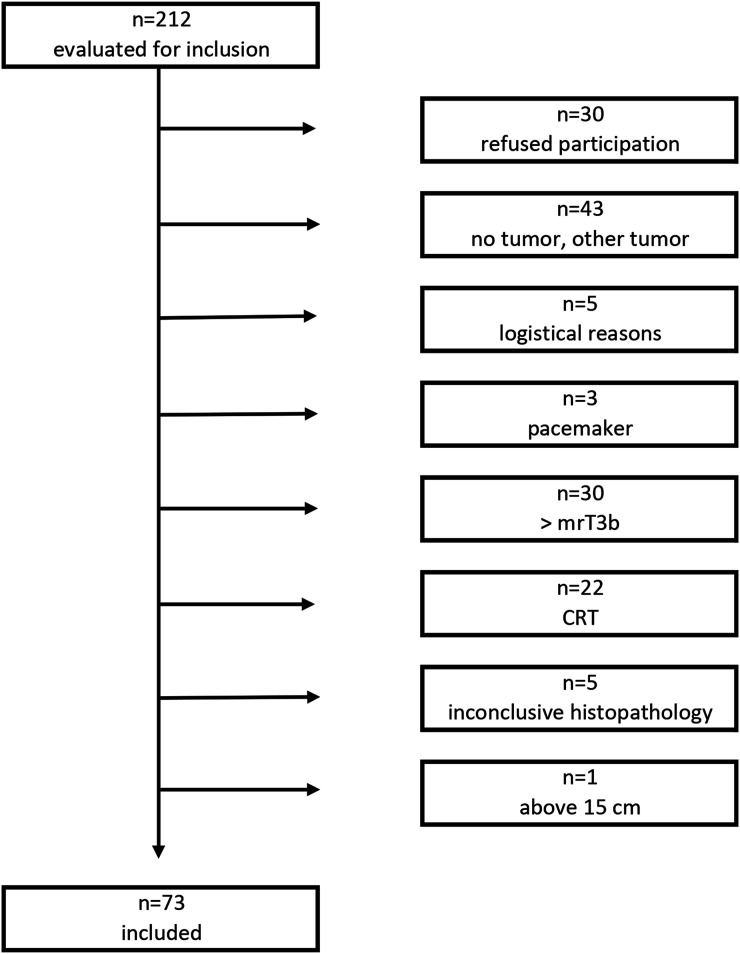
Flowchart showing inclusion and exclusion. Consecutive evaluation of all patients referred to our institution for primary treatment of rectal tumors.

From January 2016 to July 2017, we prospectively included all patients referred to our institution for newly diagnosed rectal tumors who met inclusion criteria. In total, 73 patients were enrolled in the study (Figure 1).

Inclusion criteria were rectal polyps and tumors more than 1.5 cm in size and within 15 cm of the anal verge at endoscopy. Exclusion criteria were age under 18 years, any contraindication to perform MRI, T-stage evaluated by MRI to be > T3b, preoperative chemoradiotherapy, previous surgery, and inconclusive histopathology report for T-stage.

### MRI technique

Bowel cleansing with a rectal micro-enema, 5 mL Bisacodyl 10 mg/ml (Toilax®, Orion), was performed one hour before the examination. If not contraindicated, all patients received 1 mg glucagon (Glucagon®, Novo Nordisk) i.m prior to the examination and 20–40 mg of butylscopolamine (Buscopan®, Sanofi-Aventis) i.v during the examination to reduce bowel movement artifacts.

The MRI examinations were performed on a Philips Achieva 1.5 T (Philips Medical Systems, Einthoven, the Netherlands) with a 32-channel cardiac coil or a Siemens Aera 1.5 T (Siemens Healthineers, Erlangen, Germany) with a body 30 coil. From the promontory to the anal verge, an axial 3D T2 weighted sequence (T2W) with sagittal and coronal reformats was acquired. The sequence was close to isotropic and allowed post-processing of multiplanar reformats in any direction. At tumor height, two high-resolution 2D T2W turbo spin-echo images were acquired, one perpendicular and one parallel to the long axis of the rectum and tumor. Diffusion-weighted imaging was performed in two plans, one axial full field of view from the promontory to the anal verge and one high resolution angulated parallel to the long tumor axis, identical to the angulation plane of the high-resolution 2D T2W turbo spin-echo (Supplemental Table 1).

### Image analysis

Two radiologists with 20 (EV, R1) and 13 (BH, R2) years of experience in abdominal MRI independently interpreted the anonymized study MR-images on a multimodality reading platform (syngo.via VB30®, Siemens Healthineers). R1 had five years of experience reading MRI of ERC, whereas R2 received an introduction before study start. The readers were informed about the presence and location of a rectal tumor but otherwise blinded to all clinical information. To minimize bias from an initial learning curve and align the interpretation of study-specific variables, the two readers reviewed 20 representative cases in a separate training session.

### Tumor

As far as possible, we registered the depth of the invasive tumor front into the superficial (sm1), middle (sm2), or deeper (sm3) part of submucosa according to Kikuchi.^5^

**Figure 2.**
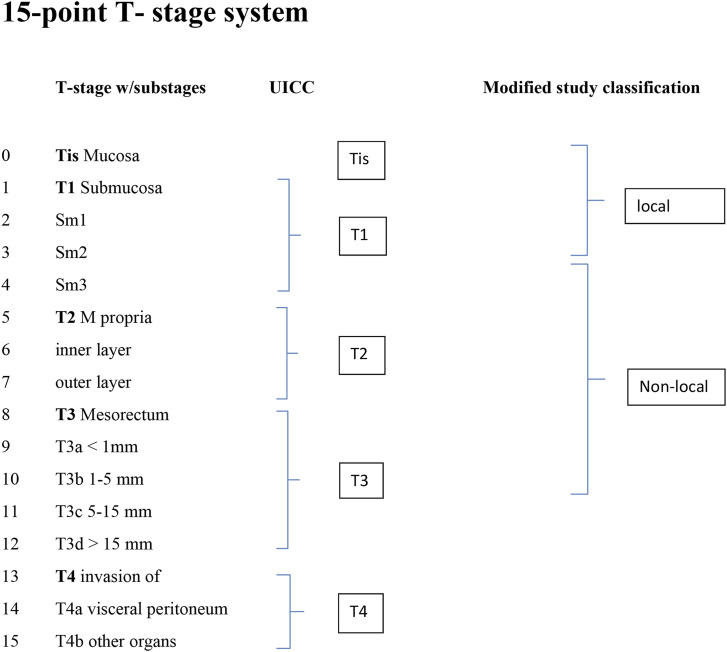
Schematic presentation of the 15-point T-stage system showing the relation to the classification system of UICC and the modified study classification.

**Figure 3.**
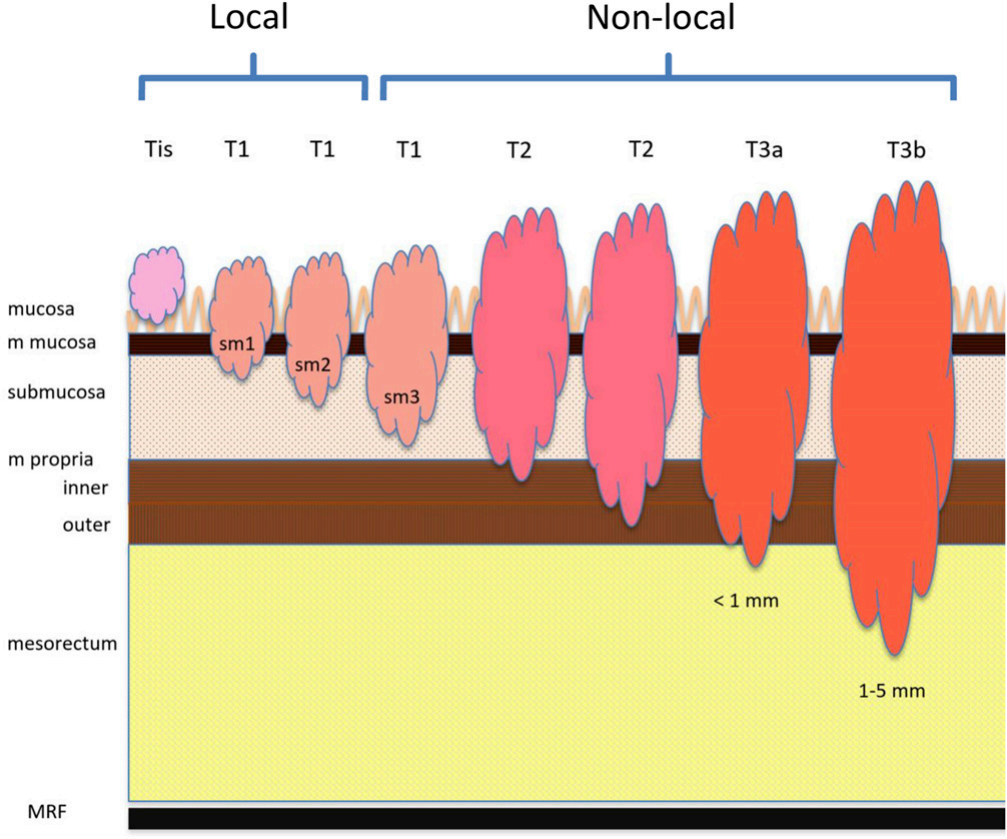
Figure visualizing the rectal wall, the level of tumor invasion, and the division in the modified classification system. MRF, mesorectal fascia.

To test the modified classification system, the T-stage of each tumor was assigned to a 15-point scale derived from the UICC TNM 8 classification system^6^ and expanded with subclasses for T1 (sm1, sm2, and sm3), T2 (inner and outer muscular layers), and T3 (T3a–T3d) (Figure 2). To stratify the tumors as appropriate for LE, adenomas and tumors with minor invasion of submucosa (Tis–T1sm2) were assigned to the group “local” and all more advanced tumors (T1sm3–T3b) to the group “non-local” (Figure 3). For comparison, the tumors also were classified according to UICC TNM8. Tumors not detected by the readers were classified as T0 and registered in the local group.

### Lymph nodes

The lymph node status was not the main aim of this study and not part of the blinded reader study. Data were derived from the clinical MRI reports representing a consensus reading of two experienced radiologists. Lymph nodes were considered benign if homogeneous with bright signal and well-defined margins on T2W. They were assessed as indeterminate if well-defined but with some degree of inhomogeneity and intermediate signal on T2W.^7^ Finally, nodes with definite inhomogeneity, low signal on T2W, and/or irregular margins were classified as malignant. Node size was not part of the classification criteria. The size of malignant nodes and nodal tumor deposits was measured at histopathology.

### Reference standard

All patients underwent surgical resection, and histopathology of the resected specimen was used as the reference standard for T-staging. For nodal staging, the reference standard was the resection specimen for all patients undergoing major resection and follow-up for patients treated with LE.

### Statistical analyses

All statistical analyses were performed using Stata (Statistical Software: Release 16. College Station, TX: StataCorp LLC). The diagnostic performance in terms of sensitivity, specificity, positive predictive value (PPV), and negative predictive value (NPP) was calculated using the Stata module diagt.^8^

Kappa values for inter- and intrareader agreement were calculated.

Intrareader agreement was calculated for R1, who had read 61 of the cases as part of routine clinical workup and later under study settings. The reading sessions were performed more than two years apart. Kappa values were interpreted according to Altman; κ < 0.20 poor, κ of 0.21–0.40 as fair, κ of 0.41–0.60 as moderate, κ of 0.61–0.80 as good, and κ of 0.81–1.00 as excellent agreement.

## Results

In total, 73 patients were included. Patient, tumor, lymph node, and treatment characteristics are shown in Table 1.

**Table 1.** Patient, tumor, lymph node, and treatment characteristics.

Variable	*n* (%)/Median (range)
Age	67 (36–90)
Sex	
Men	37 (51)
Women	36 (49)
Tumor size	
Tumor length local, mm	35 (15–120)
Tumor length non-local, mm	38 (20–68)
Location tumor	
Low rectum, 0–5 cm from anal verge	35 (48)
Midrectum, >5–10 cm from anal verge	32 (44)
Upper rectum, >10–15 cm from anal verge	6 (8)
Histopathology, benign vs malignant	
Benign (Tis)	38 (52)
Malignant (>Tis)	35 (48)
Histopathology, local vs non-local	
Local (Tis-T1sm2)	43 (59)
Non-local (T1sm3-T3b)	30 (41)
Lymph nodes	
Resected per patient	16 (3–34)
Size malignant node when tumor ≤ T2, mm	2.8 (2–3.3)
Size nodal tumor deposit when tumor ≤ T2, mm	1.7 (0.3–2.5)
Size malignant node when tumor ≥ T3, mm	5 (2–15)
Surgery	
Time-interval MRI to surgery, presumed benign tumors^a^, days	75 (9–231)
Time-interval MRI to surgery, presumed malignant tumors^a^, days	19 (1–126)
Major resection	29 (40)
Local excision LE	44 (60)
Completion surgery after LE^b^	5/44 (11)
Recurrence after LE, salvage surgery	2/41 (5)
Local tumor treated with major resection^c^	3/43 (7)

^a^Time intervals are displayed separately for preoperative clinical benign or malignant tumors because presumed adenomas were scheduled for a longer interval between MRI and surgery due to low capacity for surgery.

^b^In two patients, completion surgery was indicated but not performed due to severe comorbidity.

^c^Major resection performed in all three patients because of technical reasons.

### Operations and histopathology

LE was performed on 60% (44/73) of the patients. There were 59% (43/73) local tumors, of which 86% (37/43) were Tis and 14% (6/43) were T1sm1-sm2.

### Diagnostic performance

**Figure 4.**
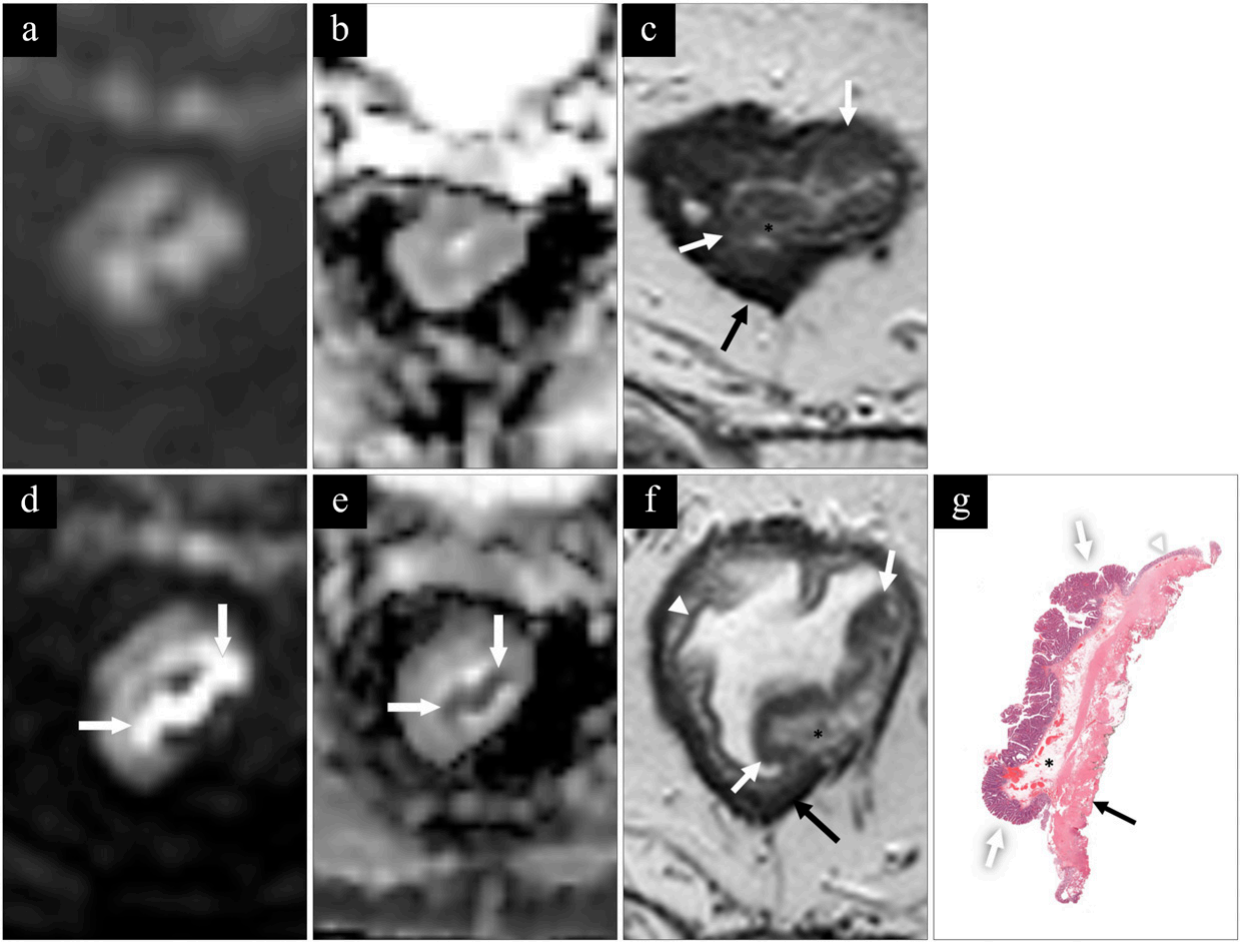
MRI and histopathology of a benign tumor (between white arrows). First row (a)–(c) without and second row (d)–(f) with rectal micro-enema. (a) and (d) Diffusion-weighted imaging (DWI) with b-1000; (b) and (e) ADC-map; (c) and (f) perpendicular high-resolution T2 TSE. There is no visible tumor infiltration in submucosa (asterisk), a finding best displayed on the T2 image after rectal micro-enema and confirmed at histopathology (g). Mucosa (white arrowhead) and muscularis propria (black arrow). The tumor is difficult to identify on DWI without rectal micro-enema while showing clear diffusion restriction after applying the rectal micro-enema.

**Figure 5.**
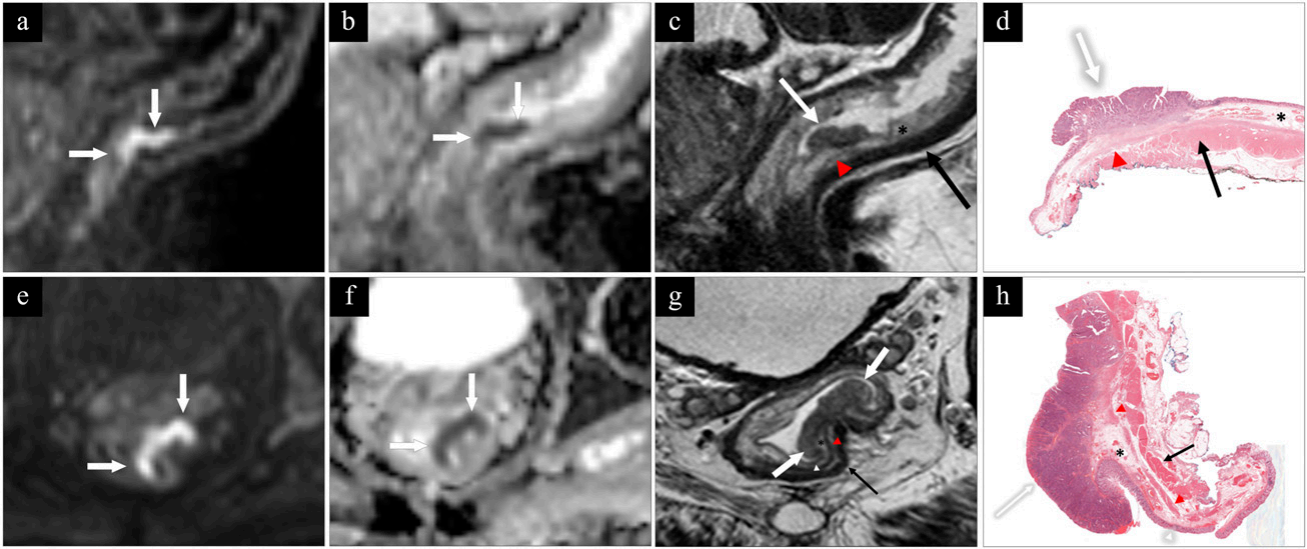
First row, pT1sm3 tumor (white arrow). Note the traction of the thin inner layer (red arrowhead) of muscularis propria towards tumor base. Submucosa (asterisk) and muscularis propria (black arrow). Second row, pT2 tumor (white arrows) The inner layer of muscularis propria (red arrowhead) is pulled against tumor base and separated from the outer layer (black arrow). The tumor is infiltrating the inner layer but not the outer layer of muscularis propria. Mucosa (white arrowhead) and submucosa (asterisk). (a) and (e) Diffusion-weighted imaging (DWI) with b-1000; (b) and (f) ADC-map; (c) sagittal 3D T2 SPACE; (g) perpendicular high-resolution T2 TSE; and (d) and (h) histopathology.

The sensitivity, specificity, PPV, NPV, and AUC to identify local tumors for R1 were 93%, 90%, 93%, 90%, and 0.92 and for R2 86%, 83%, 88%, 81%, and 0.85 (Tables 2 and 3). Examples of MRI images and histopathology are shown in Figures 4 and 5.

**Table 2.** Contingency table comparing the diagnostic performance for R1 and R2 with histopathology using the modified classification system.

	Histopathology									Total
	Tis	sm1	sm2	sm3	T2in	T2out	T3a	T3b	T3c	
Reader 1										
Local (Tis-T1sm2)	35	3	2	3	0	0	0	0	0	43
Non-local (T1sm3-T3c)	3	0	0	5	7	6	2	5	2	30
Total	38	3	2	8	7	6	2	5	2	73
Reader 2										
Local (Tis-T1sm2)	32	3	2	5^a^	0	0	0	0	0	42
Non-local (T1sm3-T3c)	6	0	0	3	7	6	2	5	2	31
Total	38	3	2	8	7	6	2	5	2	73

Sm1, sm2, and sm3, submucosal staging after Kikuchi; T2in, inner circular layer of m propria; T2out, outer longitudinal layer of m propria.

^a^One tumor (pT1sm3) not identified by R2 and registered as T0 and local.

**Table 3.** Diagnostic performance achieved by R1 and R2 for the modified classification system and the UICC TNM8 system.

	Sens % (CI), *n*	Spec % (CI), *n*	PPV % (CI)	NPV % (CI)	AUC (CI)
Reader 1 local non-local					
Local	93 (81–99), 40/43	90 (74–98), 27/30	93 (81–96)	90 (74–98)	0.92 (0.85–0.98)
Non-local	90 (74–98), 27/30	93 (81–96), 40/43	90 (74–98)	93 (81–99)	0.92 (0.85–0.98)
Reader 2 local non-local					
Local	86 (72–95), 37/43	83 (65–94), 25/30	88 (74–96)	81 (63–93)	0.85 (0.76–0.93)
Non-local	83 (65–94), 25/30	86 (72–95), 37/43	81 (63–93)	88 (74–96)	0.85 (0.76–0.93)
Reader 1 UICC TNM8					
Reader 1 Tis	80 (63–90), 30/38	80 (63–92), 28/35	81 (65–92)	78 (61–90)	0.79 (0.70–0.89)
Reader 1 T1	23 (5–54), 3/13	85 (73–93), 51/60	25 (6–57)	84 (72–92)	0.54 (0.41–0.67)
Reader 1 T2	62 (32–86), 8/13	95 (86–99), 57/60	73 (39–94)	92 (82–97)	0.78 (0.64–0.92)
Reader 1 T3	78 (40–97) 7/9	91 (81–96), 58/64	54 (25–81)	97 (89–100)	0.84 (0.69–0.99)
Reader 2 UICC TNM8					
Reader 2 Tis	61 (43–76), 23/38	86 (70–95), 29/35	82 (63–94)	67 (51–80)	0.72 (0.62–0.82)
Reader 2 T1	39 (14–68), 5/13	75 (62–85), 45/60	25 (9–49)	85 (72–93)	0.57 (0.42–0.72)
Reader 2 T2	39 (14–68), 5/13	88 (77- 95), 53/60	42 (15–72)	87 (76–94)	0.63 (0.49–0.78)
Reader 2 T3	78 (40–97), 7/9	92 (83–97), 59/64	58 (28–85)	97 (89–100)	0.85 (0.70–1.0)

R1 overstaged histopathological-verified local tumors as non-local in 7% (3/43) and R2 in 14% (6/43). R1 understaged 10% (3/30) and R2 17% (5/30) of the non-local tumors. Implementation of the data from the 15-point scale in the UICC TNM 8 system is displayed in Table 3 and Supplemental Table 2.

### Lymph nodes

**Figure 6.**
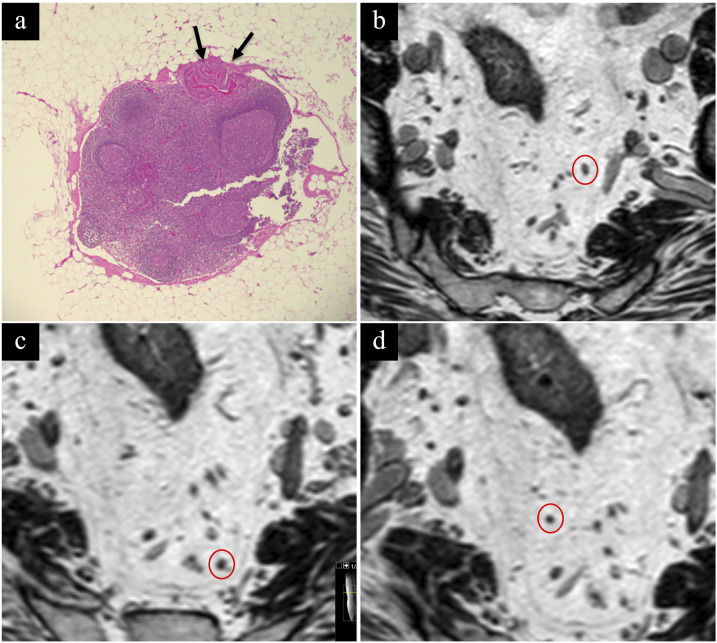
Technical limitations of MRI. (a) Histopathology showing a 2 mm lymph node with a 0.5 mm tumor deposit (black arrows) not detected by MRI. A certain assignment on the corresponding MRI images was not possible. However, possible lymph node locations are marked (red circles) on (b), (c), and (d).

Histopathologic evaluation of lymph nodes was available for 47% (34/73) of the patients, either treated primarily with major resection or subsequently with completion surgery or salvage surgery. The sensitivity, specificity, PPV, and NPV for malignant lymph nodes when regarding the indeterminate nodes as benign were 25%, 99%, 67%, and 91% versus 50%, 90%, 36%, and 94% when regarded as malignant, respectively. There was only one false-positive node when interpreting the indeterminate nodes as benign versus seven when interpreted as malignant. In total, there were eleven malignant nodes found in eight patients. For tumors ≤T2, all metastatic lymph nodes or nodal tumor deposits (*n* = 4) were smaller than 3 mm on histopathology (Figure 6). Indeterminate nodes were reported in 11.0% (8/73) of the patients, with only two having malignant nodes at histopathology.

### Inter- and intrareader agreement

In differentiating local tumors from non-local tumors, the readers achieved a kappa value of 0.69 for interreader agreement and R1 a kappa value of 0.80 for intrareader agreement.

## Discussion

In this study, MRI supplemented with a pre-procedure rectal micro-enema and concurrent use of a modified staging system achieved good accuracy in identifying tumors suitable for local excision. In patients with rectal tumor stage ≤ T2, all malignant lymph nodes or nodal tumor deposits were ≤3 mm, which is regarded as too small for characterization with MRI.^9^

There is international consensus that LE is curative in most patients with benign lesions and T1sm1 cancer. In contrast, major resection is necessary for curative treatment of T1sm3 cancers due to a 15%–20% prevalence of lymph node metastases. Guidelines, however, differ concerning the recommendations for treatment of T1sm2 cancers.^10^ Overstaging a local tumor can lead to an unnecessary rectal amputation. In contrast, undertreatment of a non-local tumor with LE can be rescued by completion surgery, probably without significant deterioration of clinical outcome. Accordingly, T1sm2 was chosen as the ideal preoperative cutoff stage for LE, accepting the risk for some completion surgeries.

For local tumors, R1 and R2 achieved a sensitivity of 93% and 86 % and specificity of 90% and 83%, respectively. This is a result somewhere in between the pooled results found in the meta-analysis by Puli et al. ^11^ with a sensitivity of 97% and specificity of 96%, and O’Connell et al.^4^ with a sensitivity of 81% and specificity of 85%, both investigating the diagnostic performance of ERUS for adenomas. Direct comparison, though, is problematic as the T-stage distribution is different. Studies found more appropriate for comparison are single-center studies of ERUS providing detailed data on diagnostic performance, making it possible to evaluate the combination of Tis and T1sm1-sm2 as one group or simply the fusion of Tis and T1 in one group.^12–17^ To make comparison more exact and focus on the early stages, data from these ERUS studies and our study were recalculated after excluding tumors >T2 (Table 4). The results from our study are in line with these ERUS studies regarding both diagnostic accuracies and rates of overstaging.

**Table 4.** Diagnostic performance of ERUS and MRI studies using T-stage selections close to our study classification system to identify tumors suitable for LE. Tumors > pT2 are excluded.

Author	T-stages	Sens % (CI)	Spec % (CI)	PPV % (CI)	NPV % (CI)	Over-staging % (*n*/n-tot)	pTis % (*n*)	pT1 % (*n*)	n-Tot
ERUS									
Akasu	Tis-Tsm1	88 (75–96)	96 (89–99)	88 (76–95)	96 (90–98)	12 (5/43)	23 (35)	29 (45)	154
Santoro	Tis-Tsm1	95 (89–99)	95 (75–100)	99 (94–100)	79 (62–90)	5 (5/106)	65 (82)	32 (40)	126
Restivo	Tis-T1	87 (77–93)	97 (85–100)	99 (91–100)	76 (64–84)	13 (11/82)	31 (37)	38 (45)	117
Zorcolo	Tis-T1	95 (87–99)	88 (47–100)	98 (91–100)	70 (43–88)	5 (3/63)	65 (46)	17	71
Patel	Tis-T1	71 (56–83)	100 (40–100)	100	22 (16–31)	27 (14/52)	(48^a^)	^a^	52
Doornebosch	Tis-T1	95 (92–98)	62 (32–86)	97 (95–99)	47 (29–66)	5 (9/196)	80 (166)	14 (30)	209
MRI									
Reader 1	Tis-T1sm2	93 (81–99)	86 (64–97)	93 (82–97)	86 (67–95)	7 (3/43)	58 (37)	22 (14)	64
Reader 2	Tis-T1sm2	86 (72–95)	76 (53–92)	88 (77–94)	73 (55–85)	14 (6/43)	58 (37)	22 (14)	64
Patel	Tis-T1	70 (54–83)	100 (40–100)	100	25 (17–35)	27 (12/44)	(40^b^)	^b^	44
Balyasnikova	Tis-T1sm2	71 (42–92)	90 (74–98)	77 (52–91)	88 (75–94)	29 (4/14)	9 4	42 (19)	45

^**a**^48 Tis and T1 registered as one group.

^b^40 Tis and T1 registered as one group. Sens, sensitivity; spec, specificity; PPV, positive predictive value; NPV, negative predictive value; pTis, Tis confirmed by histopathology; pT1, T1 confirmed by histopathology; *n*, number of patients; n-tot, number of all patients.

For MRI, the evidence for these early stages is weak. Oien et al. describe a sensitivity of 18% and a specificity of 91% with overstaging of 84% for adenomas, and sensitivity of 58% and specificity of 56% for T1 tumors.^18^ We used a similar T-stage cutoff for accepting LE as Balyasnikova et al.,^19^ but the T-stage distribution in their series was different containing few adenomas, making comparison challenging (Table 4).

The diagnostic performance of the classic UICC T-stages was poor, especially for T1 and T2 tumors. This is a known weakness for both ERUS and MRI, with reported sensitivities for ERUS for T1 from 17% in single-center studies to pooled sensitivities in meta-analysis from 50% to 88%.^3,4,18^ We reviewed the histopathology of all the T1 cancers, and most errors are due to understaging microscopic invasion (<0.4 mm) into the submucosa or overstaging T1sm3 tumors extending close to but not infiltrating muscularis propria. In addition, the tumors’ invasive front was often surrounded by fibrosis and inflammatory cells, which at this microscopic level cannot be discriminated from tumor on MRI. As the modified classification system achieved higher diagnostic performance and corresponds better to the recommendations for LE, it may be more appropriate than the UICC system in the preoperative stratification of ERC.

To avoid unnecessary major resection, the most important measure is to keep overstaging of local tumors as low as possible. In our study, local tumors were overstaged in 7% (3/43) by R1 and 14% (6/43) by R2. This is in line with comparable ERUS studies (Table 4), and both modalities still harbor a considerable risk for overtreating patients. To keep overtreating at a low rate, LE should be performed as an excision biopsy when in doubt of the depth of early invasion.

There was a good inter- and intrareader agreement with kappa values of 0.69 and 0.80, respectively; this is in the same range as similar studies confirming the robustness of the method.^19^

The accuracy for staging lymph nodes in rectal cancer is poor to moderate, with a tendency of overstaging.^20^ International MRI guidelines recommend combining size and morphological criteria to differentiate malignant nodes.^2^ However, morphological criteria such as heterogeneity and irregular border are not exact measures, and there will be a sliding transition from benign to definite malignant, creating a group of indeterminate nodes.^21^ By regarding the indeterminate nodes as benign, we achieved a high specificity of 99% with only one false-positive node but at the cost of a very low sensitivity of only 25% and eight false-negative nodes. Of the eight false-negative nodes, five nodes or nodal tumor deposits were <3 mm and too small for characterization with MRI.

In this study, all malignant lymph nodes or nodal tumor deposits in tumors ≤ T2 were too small to be characterized with MRI, a finding also reported by Landmann et al.^22^ Even though the numbers are too small to make any conclusions, this may indicate that malignant lymph nodes in ERC often are too small for preoperative imaging.

There are some limitations to this study. The patient sample was limited due to our single-center setting, yet the confidence intervals for sensitivity are comparable with similar studies.^12,14,16,17,19^ The study was conducted in an expert center by two readers experienced in advanced rectal tumors, and the diagnostic performance achieved may not be representative for daily life praxis with less experienced readers, an effect described for ERUS by Ashraf et al.^23^ Several studies have shown the importance of experience and the existence of a learning curve for ERUS, a situation probably equivalent for MRI as interpreting MRI with regard to ERC demands different knowledge compared with conventional staging of advanced tumors.^24,25^ Future research should investigate the impact of training for readers of varying experience levels. A notable limitation was the long delay of up to 6 months between MRI and surgery due to low capacity for operation of presumed adenomas. Using tumor growth-rate estimations suggested by Burke et al,^26^ this may have led to a shift from a local to a non-local tumor for one patient only. The tumor was staged as Tis by both readers, but histopathology showed T1sm3, and the tumor may have been incorrectly registered as understaged by MRI.

Imaging was done on 1.5 T MRI systems, and higher field strengths like 3.0 T might have improved special resolution. The readers in this study demonstrated higher diagnostic performance than in previously reported MRI studies. The reason is probably multifactorial, including technical issues such as new sequences like 3D T2 and DWI, training, and experience of the readers for MRI of ERC, or potentially patient preparation with a micro-enema. However, the specific impact of the micro-enema on diagnostic outcomes remains inconclusive due to study design and warrants further exploration in dedicated studies.

In conclusion, MRI supplemented with a pre-procedure rectal micro-enema and concurrent use of a modified staging system achieved good accuracy to identify tumors suitable for LE.

The rate of overstaging of local tumors was in the range of results reported in previous ERUS studies, and the risk for overtreatment still is an issue of concern. For T1 and T2 tumors, metastatic lymph nodes were too small to be characterized by MRI.

## Supplemental Material

Supplemental Material-Early rectal cancer: The diagnostic performance of MRI supplemented with a rectal micro-enema and a modified staging system to identify tumors eligible for local excision Supplemental Material for Early rectal cancer: The diagnostic performance of MRI supplemented with a rectal micro-enema and a modified staging system to identify tumors eligible for local excision by Ellen Viktil, Bettina Andrea Hanekamp, Arild Nesbakken, Else Marit Løberg, Ole Helmer Sjo, Anne Negård, Johann Baptist Dormagen, and Anselm Schulz in Acta Radiologica Open.

## References

[1] Morino M Risio M Bach S , et al. Early rectal cancer: the European Association for Endoscopic Surgery (EAES) clinical consensus conference. Surg Endosc 2015; 29: 755–773. 25609317 10.1007/s00464-015-4067-3

[2] Beets-Tan RGH Lambregts DMJ Maas M , et al. Magnetic resonance imaging for clinical management of rectal cancer: updated recommendations from the 2016 European Society of Gastrointestinal and Abdominal Radiology (ESGAR) consensus meeting. Eur Radiol 2018; 28: 1465–1475. 29043428 10.1007/s00330-017-5026-2 PMC5834554

[3] Puli SR Bechtold ML Reddy JB , et al. How good is endoscopic ultrasound in differentiating various T stages of rectal cancer? Meta-analysis and systematic review. Ann Surg Oncol 2009; 16: 254–265. 19018597 10.1245/s10434-008-0231-5

[4] O’Connell E Galvin R McNamara DA , et al. The utility of preoperative radiological evaluation of early rectal neoplasia: a systematic review and meta-analysis. Colorectal Dis 2020; 22: 1076–1084. 32052545 10.1111/codi.15015

[5] Kikuchi R Takano M Takagi K , et al. Management of early invasive colorectal cancer. Risk of recurrence and clinical guidelines. Dis Colon Rectum 1995; 38: 1286–1295. 7497841 10.1007/BF02049154

[6] Brierly JD . UICC TNM classification of malignant tumours. 8th ed. New Jersey, NJ: Wiley-Blackwell, 2016.

[7] Helsedirektoratet. Nasjonalt handlingsprogram med retningslinjer for diagnostikk, behandling og oppfølging av kreft i tykktarm og endetarm, 6. edition. 2019.

[8] Seed P . DIAGT: Stata module to report summary statistics for diagnostic tests compared to true disease status. Boston: Boston College Department of Economics, 2010. https://ideas.repec.org/c/boc/bocode/s423401.html

[9] Brown G Richards CJ Bourne MW , et al. Morphologic predictors of lymph node status in rectal cancer with use of high-spatial-resolution MR imaging with histopathologic comparison. Radiology 2003; 227: 371–377. 12732695 10.1148/radiol.2272011747

[10] Borstlap WAA van Oostendorp SE Klaver CEL , et al. Organ preservation in rectal cancer: a synopsis of current guidelines. Colorectal Dis 2017; 43: 23. 10.1111/codi.13960 29136328

[11] Puli SR Bechtold ML Reddy JB , et al. Can endoscopic ultrasound predict early rectal cancers that can be resected endoscopically? A meta-analysis and systematic review. Dig Dis Sci 2010; 55: 1221–1229. 19517233 10.1007/s10620-009-0862-9

[12] Restivo A Zorcolo L Marongiu L , et al. Limits of endorectal ultrasound in tailoring treatment of patients with rectal cancer. Dig Surg 2015; 32: 129–134. 25791387 10.1159/000375537

[13] Doornebosch PG Bronkhorst PJ Hop WC , et al. The role of endorectal ultrasound in therapeutic decision-making for local vs. transabdominal resection of rectal tumors. Dis Colon Rectum 2008; 51: 38–42. 18038181 10.1007/s10350-007-9104-4

[14] Zorcolo L Fantola G Cabras F , et al. Preoperative staging of patients with rectal tumors suitable for transanal endoscopic microsurgery (TEM): comparison of endorectal ultrasound and histopathologic findings. Surg Endosc 2009; 23: 1384–1389. 19263149 10.1007/s00464-009-0349-y

[15] Santoro GA Gizzi G Pellegrini L , et al. The value of high-resolution three-dimensional endorectal ultrasonography in the management of submucosal invasive rectal tumors. Dis Colon Rectum 2009; 52: 1837–1843. 19966629 10.1007/DCR.0b013e3181b16ce9

[16] Akasu T Kondo H Moriya Y , et al. Endorectal ultrasonography and treatment of early stage rectal cancer. World J Surg 2000; 24: 1061–1068. 11036283 10.1007/s002680010151

[17] Patel RK Sayers AE Kumar P , et al. The role of endorectal ultrasound and magnetic resonance imaging in the management of early rectal lesions in a tertiary center. Clin Colorectal Cancer 2014; 13: 245–250. 25301243 10.1016/j.clcc.2014.09.002

[18] Oien K Forsmo HM Rosler C , et al. Endorectal ultrasound and magnetic resonance imaging for staging of early rectal cancers: how well does it work in practice? Acta Oncol 2019; 58: S49–S54. 30736712 10.1080/0284186X.2019.1569259

[19] Balyasnikova S Read J Wotherspoon A , et al. Diagnostic accuracy of high-resolution MRI as a method to predict potentially safe endoscopic and surgical planes in patients with early rectal cancer. BMJ Open Gastroenterol 2017; 4: e000151. 10.1136/bmjgast-2017-000151 PMC5730880 29259791

[20] Al-Sukhni E Milot L Fruitman M , et al. Diagnostic accuracy of MRI for assessment of T category, lymph node metastases, and circumferential resection margin involvement in patients with rectal cancer: a systematic review and meta-analysis. Ann Surg Oncol 2012; 19: 2212–2223. 22271205 10.1245/s10434-011-2210-5

[21] Grimm P Loft MK Dam C , et al. Intra- and interobserver variability in magnetic resonance imaging measurements in rectal cancer patients. Cancers 2021; 13: 120. 34680269 10.3390/cancers13205120 PMC8534180

[22] Landmann RG Wong WD Hoepfl J , et al. Limitations of early rectal cancer nodal staging may explain failure after local excision. Dis Colon Rectum 2007; 50: 1520–1525. 17674104 10.1007/s10350-007-9019-0

[23] Ashraf S Hompes R Slater A , et al. A critical appraisal of endorectal ultrasound and transanal endoscopic microsurgery and decision-making in early rectal cancer. Colorectal Dis: The Official Journal of the Association of Coloproctology of Great Britain and Ireland 2011; 14: 821–826. 10.1111/j.1463-1318.2011.02830.x 21920011

[24] Surace A Ferrarese A Gentile V , et al. Learning curve for endorectal ultrasound in young and elderly: lights and shades. Open Med 2016; 11: 418–425. 10.1515/med-2016-0074 PMC5329861 28352830

[25] Marusch F Ptok H Sahm M , et al. Endorectal ultrasound in rectal carcinoma--do the literature results really correspond to the realities of routine clinical care? Endoscopy 2011; 43: 425–431. 21234855 10.1055/s-0030-1256111

[26] Burke JR Brown P Quyn A , et al. Tumour growth rate of carcinoma of the colon and rectum: retrospective cohort study. BJS Open 2020; 198: 151–158. 10.1002/bjs5.50355 PMC8370463 32996713

