# Supplementary material for: Early rectal cancer: The diagnostic performance of MRI supplemented with a rectal micro-enema and a modified staging system to identify tumors eligible for local excision
Source: Acta Radiol Open. 2024 Apr 18;13(5):20584601241241523. doi: 10.1177/20584601241241523 (PMC11027598; doi:10.1177/20584601241241523)
Supplement: Supplemental Material-Early rectal cancer: The diagnostic performance of MRI supplemented with a rectal micro-enema and a modified staging system to identify tumors eligible for local excision [file sj-pdf-1-arr-10.1177_20584601241241523.pdf]

Supplementary Table 1 MR imaging acquisition parameters for Philips Achieva 1.5 T and Siemens Aera 1.5T

**1.5 Tesla (Phillips Achiva)**	**T2W TSE**	**T2W TSE**	**T2 3D VISTA**[a]	**DWI**	**DWI**	**T1 3D VISTA**
Imaging planes	Oblique axial[b]	Oblique parallel[c]	Axial with sagittal and coronal reformates	Oblique parallel	Axial	Coronal
Repetition time/Echo time(msec)	4695/90	4695/90	1175/85	1262/83	2808/69	250/14
Bandwidth (Hz/pixel)	195	195	445	2234	2629	430
Field of view (mm)	160 x 160	160 x 160	289/190	20 x 20	330 x 330	460 x 460
Acquisition Matrix	256/224	256/224	292/252	80 x 117	112 x 107	3841/382
Section thickness/gap (mm)	3/0.3	3/0.3	1/-0.5	5/1	5/1	1.2/-0.6
Number of signals acquired/b-value	6	6	1	8/b-0 8/b-500 8/b-1000	3/b-0 3/b-500 3/b-1000	2
**1.5 Tesla (Siemens Aera)**	**T2W TSE**	**T2W TSE**	**T2 3D SPACE**[d]	**DWI**	**DWI**	**T1 3D SPACE**
Imaging planes	Oblique axial	Oblique parallel	Axial with sagittal and coronal reformates	Oblique parallel	Axial	Coronal
Repetition time/Echo time(msec)	4250/82	4250/82	1300/90	3400/69	3800/65	350/19
Bandwidth (Hz/pixel)	225	225	698	1445	1812	345
Field of view (mm)	200x200	200x200	256x256	200x200	368x248	384x384
Acquisition Matrix	320x320	320x320	256x256	128x128	184/124	384x384
Section thickness/gap (mm)	3/0.3	3/0.3	1.1/0	5/0	4/0.8	1.3/0
Number of signals	3	2	1.4	3/b-50 4/b-300	4/b-500 9/b-1000	1.4

acquired/b-value	11/b-700 Calculated b-1500

Supplementary Table 2. Contingency table comparing the diagnostic performance for R1 and R2 with histopathology using the UICC TNM8 system.

	Histopathology				
	**Tis**	**T1**	**T2**	**T3**	**Total**
**Reader 1**					
**Tis**	29	7	0	0	36
**T1**	9	3	0	1	13
**T2**	0	2	8	1	11
**T3**	0	1	5	7	13
**Total**	38	13	13	9	73
**Reader 2**					
**Tis**	23	6[a]	0	0	29
**T1**	12	5	3	0	20
**T2**	3	2	5	2	12
**T3**	0	0	5	7	12
**Total**	38	13	13	9	73

[a] One tumor T1sm3 not identified by R2 and registered as T0 and benign.
